# Addressing threats to validity in large dataset research

**DOI:** 10.1371/journal.pmed.1005282

**Published:** 2026-11-02

**Authors:** Wonsuk Yoo, John P. A. Ioannidis

**Affiliations:** 1 School of Nursing and Health Studies, Division of Biostatistics/Bioinformatics, Department of Public Health Sciences, TCO Program, Sylvester Comprehensive Cancer Center, University of Miami, Coral Gables, Florida, United States of America; 2 Meta-Research Innovation Center at Stanford (METRICS), Stanford University, California, United States of America; 3 Stanford Prevention Research Center, Department of Medicine, Stanford University School of Medicine, Stanford, California, United States of America; 4 Department of Epidemiology and Population Health, Stanford University School of Medicine, Stanford, California, United States of America; 5 SDepartment of Biomedical Data Science, Stanford University School of Medicine, Stanford, California, United States of America

## Abstract

Large dataset research offers new opportunities across biomedicine, but it also faces major threats to validity. These threats are underappreciated and often not recognized or managed appropriately. We dissect here 4 major types of validity threats: (1) *P*-value inflation and trivial effects; (2) residual and differential confounding across multiple sites; (3) issues surrounding harmonization and measurement error; and (4) causal identification in the presence of rich covariates. For each of them we provide their theoretical background, empirical evidence, and simulations that showcase how they operate. We make recommendations that may offer guidance for analyses and inferences from large datasets across biomedicine. These include: deciding on the placement in a continuum of exploration versus targeted causation; evaluating both statistical significance and effect sizes; modeling site heterogeneity; treating harmonization as measurement modeling; considering causal diagrams to guide adjustment; checking covariate balance; embedding sensitivity analysis and exploration of robustness; reporting limitations structurally; considering falsification calibration of performance; considering multi-analyst approaches; enhancing sharing, openness, transparency, and documentation; and considering encompassing analyses rather than least publishable units. Improving large dataset research and adequately addressing these threats is essential for the integrity of the scientific record and eventually also for informing policy and clinical practice.

## Abstract 2

In this Guidelines and Guidance article, Wonsuk Yoo and John P.A. Ioannidis outline why research using large datasets faces threats to validity, highlighting four main statistical pitfalls and providing guidance and worked examples on how to mitigate against them.

## Introduction

In recent years, biomedical research has been revolutionized by the use of large integrated databases such as ECHO [1], SEER-Medicare [2], and UK Biobank [3], as well as electronic health record (EHR) data [4], which connect clinical, environmental, genomic, and social data on an unprecedented scale. These datasets offer greater statistical power and diversity, enabling analysis of complex relationships. However, their size and complexity also reveal limitations in traditional statistical methods that also pose escalated threats to the validity of research utilizing such large datasets. Familiar tools such as *p*-values and confidence intervals may be misleading and meaningless. As datasets grow, sources of bias may become more pronounced, prompting a reassessment of valid inference in biomedical science [5–7].

Traditional study designs were created for situations with limited data, where random error was a major concern and hypothesis-testing helped manage uncertainty [8,9]. In large datasets, random error may become tiny, but systematic error may remain or even grow. Even minor differences can produce extremely low *p*-values [6,10]. Additionally, combining data from multiple sources leads to confounding that standard adjustments can’t fully resolve [11,12]. The result is a new paradox of large data: precision without validity. Small uncertainties may be estimated with great confidence, while the underlying effect remains conceptually mis-specified. With hundreds of variables, it becomes hard to distinguish confounders, mediators, and colliders (see Box 1: Glossary). Over-adjusting may block or create causal paths, resulting in statistically correct but causally irrelevant estimates [13,14]. Harmonizing data across cohorts introduces measurement errors and information loss [15,16], which in large datasets leads to biased results with deceptively precise confidence intervals [17]. Structural biases and site differences pose further major validity threats.

**Box 1: Glossary**

**Attenuation simulation:** A simulation technique used to evaluate the extent to which measurement error attenuates an estimated association or effect.

**Bland-Altman plot:** A graphical method used to compare two measurement methods or techniques by plotting the differences between them against their averages.

**Collider:** A variable that is simultaneously influenced by two (or more) other variables; controlling for a collider should be avoided because it creates a spurious association between these other two variables.

**Completed partially DAG:** A graph that depicts an equivalence class of multiple causal DAGs (see below) that share conditional independence relationships, including directed arrows of causal relationships that are uniquely determined and identical across all valid DAGs in the equivalence class and undirected edges where the direction of causality varies across DAGs.

**Confounder:** A variable that is associated with an exposure and with an outcome but is not in the causal pathway from the exposure to the outcome; controlling for a confounder is essential to understand if there is a causal relationship.

**Directed acyclic graph (DAG):** A visual graph using arrows to show causal pathways between variables without any feedback loops.

**Hierarchical modeling:** A statistical model that accounts for data grouped in nested levels.

**Inverse probability weighting:** A method that weighs data points by the reciprocal of their treatment probability to create a synthetic sample where confounders are better balanced.

**LASSO:** A regression method for automatic variable selection that penalizes the absolute size of coefficients, shrinking to zero the least important ones and those that are highly correlated.

**Latent-variable modeling:** A modeling framework that estimates abstract, unobserved concepts using multiple directly observed variables.

**Mediator:** An intermediate variable that sits on the causal pathway from exposure to outcome.

**Multiple-indicator modeling:** A latent-variable approach that employs observed measures to represent a single underlying construct to reduce measurement error.

**Propensity score:** The probability that a subject receives a specific treatment based on their observed characteristics.

**Regression calibration:** A method that substitutes an error-prone predictor variable with its expected value which may be calculated from a validation sub-study.

**Simulation-extrapolation (SIMEX):** A simulation approach that adds baseline error to data, measures the trend of worsening bias, and then extrapolates backward to estimate the true effect without error.

**Specification curve analysis:** A method that runs every defensible alternative model combination to show how different analytical choices may affect the results.

**Stepwise regression:** An automated variable selection algorithm that adds or removes variables from a model one at a time based on some statistical criterion.

**Trimming:** The practice of dropping observations with extreme propensity scores to prevent data points with extremely high or extremely low probabilities from destabilizing the analysis.

**Vibration of effects:** The variation of a treatment effect when all possible analytical approaches, model choices, and/or sampling choices are considered.

**Weight stabilization:** A modification to inverse probability weights that multiplies them by the baseline probability of treatment, in order to avoid inflation of variance from extreme weights.

Large dataset analyses may have different purposes [18] that correspond to causal, descriptive, or predictive questions. Some problems such as confounding are relevant to causal questions, while other problems such as issues with *p*-values and measurement error affect large dataset research more broadly. Exploratory analyses would typically align mostly with descriptive estimands. However, it is relatively common for authors to indulge in causal language and spurious causal inferences even in these situations. Framing questions with clarity as to whether the pursued associations are causal or non-causal and using confidence intervals to present what associations the data and models are most compatible with (rather than whether or not there is an effect) may sometimes reduce some misinterpretations. A statistically significant result may not be necessarily clinically meaningful, and a non-significant result may not necessarily indicate no true association [19]. However, even when confidence intervals are used, persistent misinterpretations may remain a challenge [20,21].

Statistical and methodological threats to the validity of large dataset research are not just theoretical concerns for methodologists and data scientists. Large dataset research is increasingly informing medical policies and guidelines and there is a push for replacing randomized trials with the products of such large dataset analyses. Lower cost and the facility of obtaining results very quickly instead of waiting years for randomized trials to be done are touted as major advantages and often some non-randomized analyses are presented as equivalent if not superior to randomized trials [22]. However, analyses lacking validity may misinform and lead to suboptimal or detrimental health outcomes for individual patients, preventive strategies, and communities for medicine and public health decisions.

Big data debates tend to emphasize computational efficiency and accuracy over foundational issues like causation, bias, and measurement [23,24]. This paper outlines four main challenges: *p*-value inflation, confounding across sites, harmonization and measurement error, and causal identification with numerous covariates. Each challenge is explored with conceptual analysis and simulations, showing that bias often outweighs randomness in uncertainty. Along these lines, we also propose some guidance that may help make large-scale analytics more meaningful, credible, and reproducible. We use the term “large data” broadly to encompass datasets characterized by large sample size, high-dimensional variable measurement, or both. Such datasets often include extensive demographic, biological, social, and environmental information.

## *P*-value inflation and trivial effects

Hypothesis testing with *p*-values was built when data were scarce and experiments were expensive. In the large-data setting, that basic logic bends. Null hypothesis significance testing (NHST) asks, “If there were truly no effect, how surprising would these data be?”. But when the sample size is huge, almost any small departure from the null is “surprising” in the technical sense. Sampling variability shrinks, standard errors get tiny, and test statistics explode even when the underlying effect is biologically or clinically trivial. The result is *p*-value inflation, an overabundance of very small *p*-values that look persuasive but correspond to effect sizes that are too small to matter biologically or clinically [5,9]. With big data, the key question changes from "can we find it?" to "is it worth finding?"

There are at least three interrelated mechanisms behind this problem: true-but-not-worth-it effects get detected, spurious effects get detected, and too many effects may be pursued – and get detected. First, the test statistics for common models (t-tests, Wald tests, likelihood-ratio tests) grow roughly with $\sqrt{n}$, so doubling or tripling the sample size can turn a noisy, borderline association into one that is highly statistically significant for the same effect size [6,25,26].

Second, real-world large databases such as EHR-based cohorts, consortia, or multi-site studies, almost always have some model misspecification or leftover noise (e.g., unmeasured confounders, imperfect harmonization, nonlinearity). When working with very large samples, the model detects the misspecification and responds by producing low *p*-values for spurious effects.

Third, analysts frequently conduct numerous related tests across outcomes, subgroups, or exposures, as permitted by the rich data. In high-powered analyses, this combination of multiple comparisons and very small standard errors almost inevitably results in numerous statistically significant findings [5]. Correcting for multiplicity solves only part of the problem, since many true-but-not-worth-it effects and spurious effects may still survive multiplicity corrections in extremely highly powered datasets. Moreover, multiplicity corrections often fail to consider the full extent of comparisons and analytical choices engaged and thus offer subpar adjustments in statistical significance or false discovery rate. For example, while public availability of large datasets is welcome, many investigators can perform analyses in the same analytical space; even if each of them properly adjusts for multiplicity their own analyses, there is no adjustment for the massive analytical onslaught occurring by multiple teams on the same data.

Different scientific communities in different contexts may have different perceptions of what effect is worth finding and may have given different thought to this question or no thought at all. Some caveats should be acknowledged. First, effect sizes are what nature decides, not what scientists want. In many fields of scientific investigation informed by large datasets, true effect sizes may be very small. Second, many scientists may defend the pursuit of tiny effect sizes as a genuinely interesting enterprise to understand the overall architecture of risks, even without obvious applied utility. Third, combinations of many small, but genuine risks may still yield some utility. For example, the pursuit of genetic associations for complex diseases and the generation of polygenic risk scores [27] may fit these descriptions—even though final utility may still be questioned. Nevertheless, in the clinical and policy context, marginal differences are typically insufficient to meaningfully inform decision-making.

As shown in Fig 1A, repeated two-sample *t* test simulations under a fixed small effect size demonstrate that *p*-values decline systematically as sample size increases despite no change in the underlying effect magnitude. In Fig 1B, large-sample regression simulations further show that highly statistically significant *p*-values frequently occur for effect estimates near zero when standard errors are small. Finally, Fig 2 illustrates that in very large multivariable datasets, trivial true effects and spurious/confounded associations can become nearly as statistically compelling as meaningful causal effects. Together, these simulations demonstrate that in large datasets, statistical significance may increasingly reflect sample size and precision rather than effect magnitude and practical importance, let alone causal validity.

**Fig 1:**
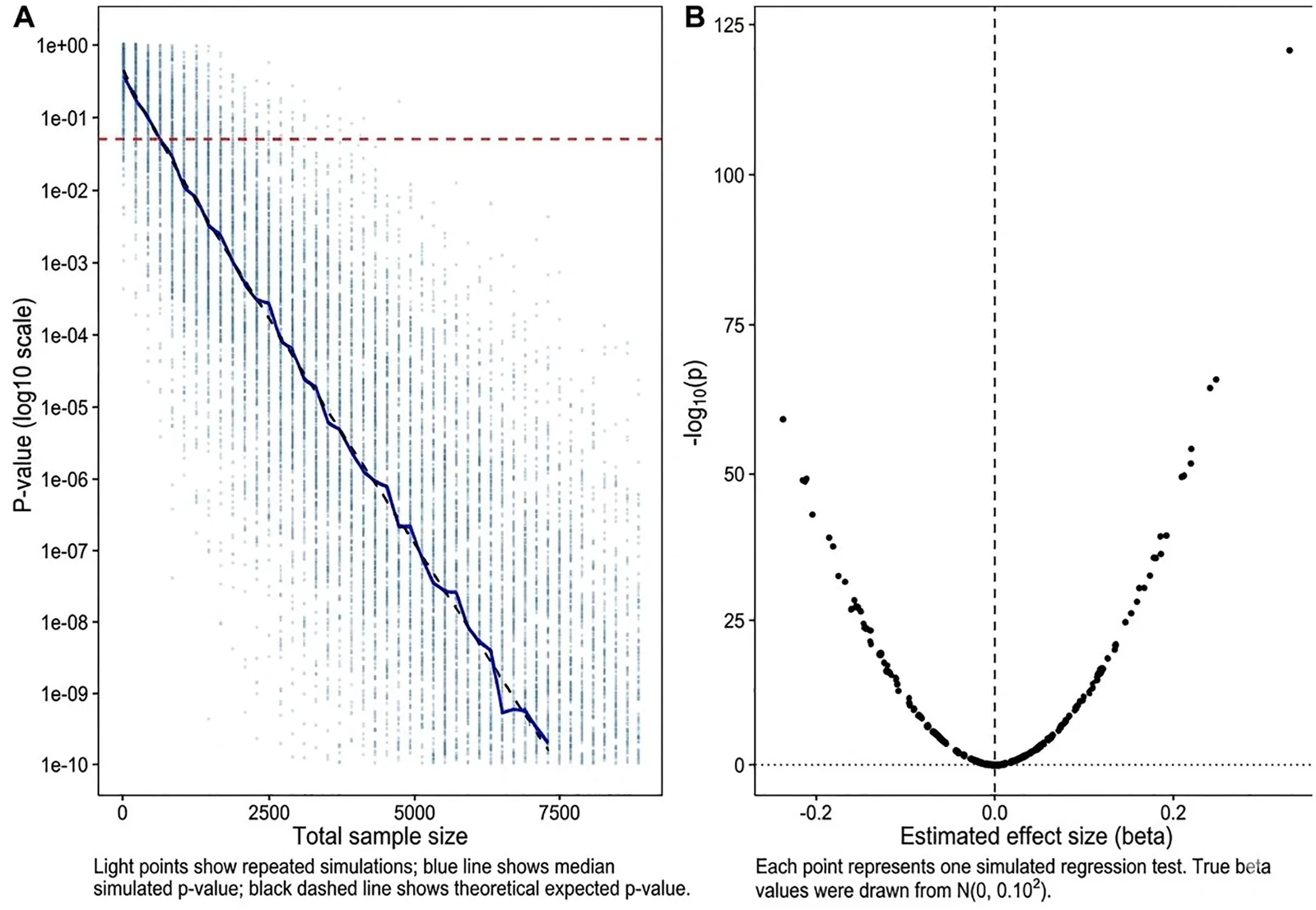
Impressive *p*-values and small effect sizes. (**A**) ***P*-value decline with increasing sample size for a small true effect.** Here, we simulated two independent normally distributed groups with equal variance (σ=1) and a constant standardized mean difference of Cohen’s d=0.15, representing a small effect size. Total sample size ranged from 100 to 9,000, equally divided between groups, and 300 independent two-sample *t* test simulations were performed at each sample size. *P*-values declined systematically as sample size increased, with sufficien*t*ly large samples producing extremely small *p*-values despite the always small underlying effect. *Take-home message: In large datasets, statistical significance may increasingly reflect how big the study is rather than substantive effect magnitude or practical importance.* (**B**) **Disconnect between effect size and statistical significance in simulated regressions.** Here, the graph illustrates the disconnect between statistical significance and effect magnitude in large-sample regression analyses. We simulated 200 independent linear regression models, each with 5,000 observations, in which the true regression coefficient for the exposure was randomly drawn from $\left(\beta_{true}\sim N(\text{0},{\text{0}.\text{1}\text{0}}^{\text{2}})\right)$, thereby generating predominantly small true effects. Outcomes were simulated according to Y=βX+ϵ, where X~N(0,1) and ϵ~N(0,1), and estimated regression coefficients with corresponding *p*-values were extracted from each fitted model. Despite the predominance of very small true effects, many results yielded highly statistically significant *p*-values. *Take-home message: Increasing sample size improves precision and statistical significance, but it does not make a small effect clinically important.*

**Fig 2:**
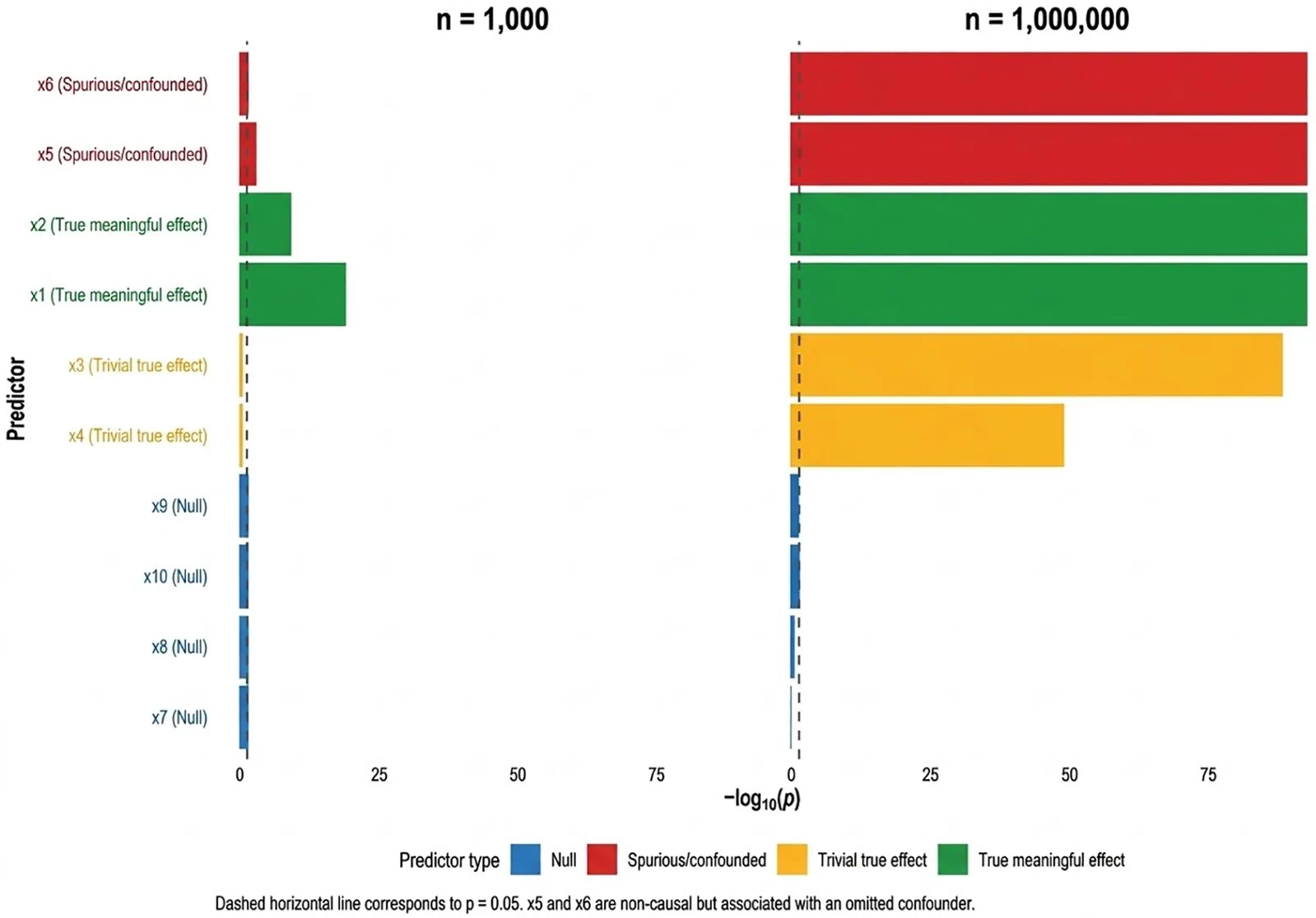
Inflation of statistical significance with increasing sample size. We simulated multivariable linear regression models under two sample size scenarios (n=1,000 and n=1,000,000) using 10 predictors representing distinct inferential scenarios. Predictors $x_{\text{1}}$ and $x_{\text{2}}$ were assigned meaningful true effects on the outcome, $x_{\text{3}}$ and $x_{\text{4}}$ had trivial but nonzero true effects, $x_{\text{5}}$ and $x_{\text{6}}$ had no causal effect but were associated with the outcome through an unmeasured confounder, and $x_{\text{7}}$–$x_{\text{1}\text{0}}$ were true null predictors. At moderate sample size, only the strongest true effects were consistently highly significant, whereas trivial and spurious associations showed weaker evidence. However, at very large sample size, trivial true effects and spurious confounded associations became nearly as statistically significant as the meaningful predictors. *Take-home message: Very large sample sizes make meaningful, trivial, and biased associations appear highly significant. I.e., in sufficiently large datasets, statistical significance may fail to distinguish clinically important effects from negligible or biased associations, thereby increasing the risk of overinterpreting trivial or noncausal findings.*

## Residual and differential confounding across sites

Large datasets often combine data from various regions, institutions, and populations. This diversity offers scientific advantages but also leads to significant residual and differential confounding, which may persist even after adjusting for typical covariates. While in some studies the region/site will not be a confounder after adjusting for individual determinants of exposure, in many others these individual adjustments are not feasible, and the site/region may be an unblocked backdoor path between exposure and outcome. Exposures, outcomes, and covariates often vary systematically depending on the site. For example, urban cohorts of children might face higher pollution, have different socioeconomic backgrounds, and access healthcare differently compared to those in rural areas. When these context-specific factors affect both exposure and outcome and are not fully accounted for by measured covariates, site-level confounding can occur, biasing aggregate associations [7,12].

The challenge becomes more pronounced when analytic models regard all sites as interchangeable and aggregate data using a single intercept. This practice of ignoring sites provenance and performing naïve pooling presumes uniformity in exposure–outcome relationships across sites. In practice, however, site-specific variations in recruitment, measurement methodologies, and unmeasured factors may lead to differential confounding, introducing bias that differs by both direction and magnitude among sites [28,29].

Conventional approaches for adjusting observed site indicators, such as incorporating a site as a fixed effect, are often insufficient because residual confounding within sites may remain and exposure distributions can differ substantially across locations. Employing random-effects or hierarchical modeling (see Box 1: Glossary) may address part of this challenge by permitting both intercepts and slopes to vary by site [11], but assumptions on the distribution of intercepts and slopes are arbitrary. Alternative methods include fixed-effect variants [30] within-site estimation followed by meta-analysis [31,32], inverse-probability weighting to balance covariate distributions, and propensity-score calibration utilizing site-specific covariates [33–36] (see Box 1: Glossary). Directed acyclic graphs (DAGs) [7,14,37–40] may help determine when a site serves as a confounder, mediator, or collider, thereby informing appropriate adjustment [7,12]. All of these approaches have their own caveats, and they have been applied only to a few published large dataset analyses to date. With increasing emphasis on large sample sizes and the impressive-looking statistical significance of results, there is often limited or no information on the site provenance of the constituent data, let alone any effort to account for it.

Given these considerations, the large dataset paradigm may perform even worse than the traditional past approach of performing relatively small studies and then trying to synthesize their results through meta-analysis. The meta-analysis may suffer more from publication and selective reporting biases that might be avoided if all the small studies from different sites and investigators were upfront combined in a single large dataset. However, the single dataset analysis will perform worse than the traditional meta-analysis if it ignores site provenance or does not use a suitable model to account for it.

Fig 3 illustrates residual and differential confounding across sites. In Fig 3A, site-specific regression models produced substantial heterogeneity in observed exposure-effect estimates despite a common true causal effect, demonstrating that residual confounding can generate marked between-site variability even when underlying biology is constant. Fig 3B further shows that with increasing site-to-site imbalance in an unmeasured confounder, the pooled estimate progressively diverges from the true effect despite unchanged sample size and apparent precision. Together, these results demonstrate that cross-site differences in unmeasured confounding can distort both site-specific and pooled estimates in multicenter observational analyses.

**Fig 3:**
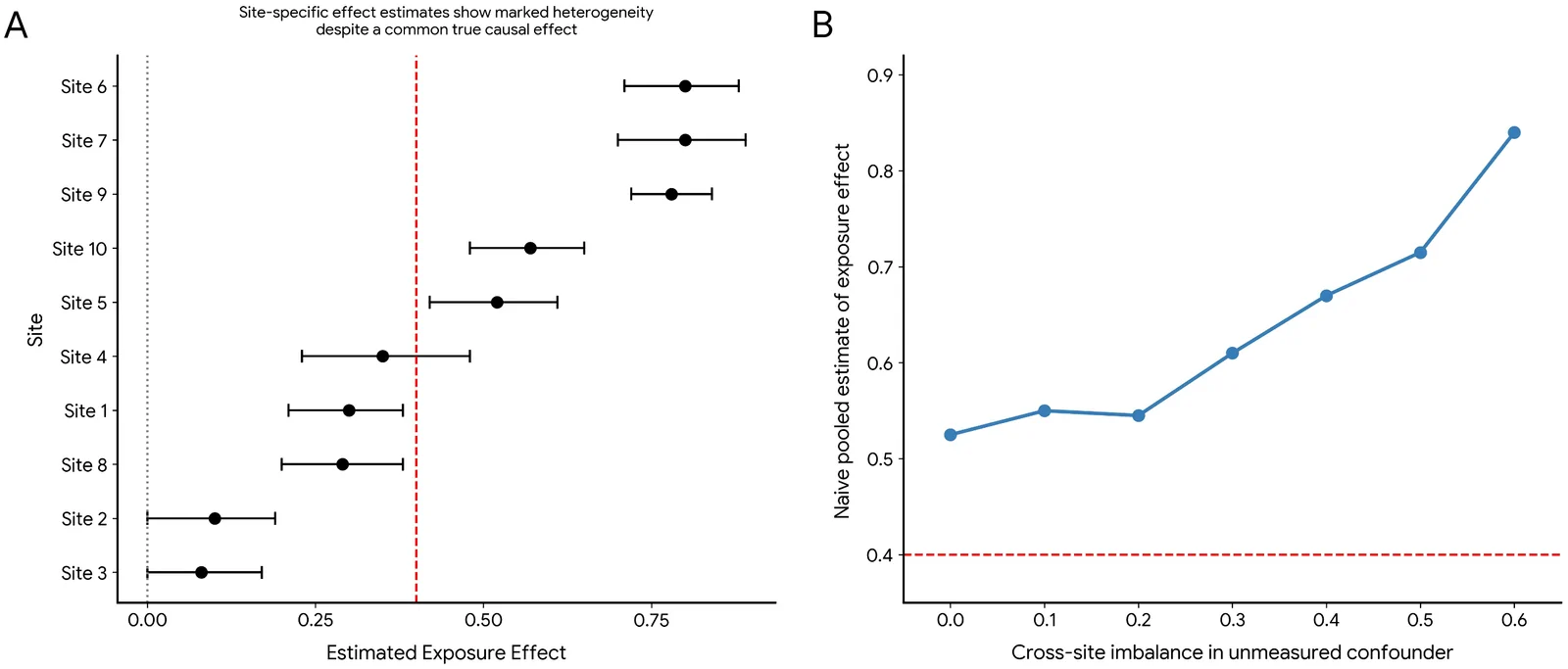
Differential confounding across sites biases estimates of effects. **(A) Differential confounding across sites despite a common true effect.** This graph illustrates residual and differential confounding across sites in multicenter observational analyses. We simulated multicenter data across 10 sites with 500 individuals per site under a common true exposure effect of β=0.40. Within each site, an unmeasured confounder influenced both exposure and outcome, but the magnitude and direction of these confounding pathways were allowed to vary across sites to represent differences in population composition, measurement practices, referral patterns, and other unrecorded factors. Site-specific regression models omitting the unmeasured confounder were then fit separately within each site. Despite an identical underlying causal effect across all sites, the estimated exposure effects varied substantially, with some sites overestimating the true effect, others attenuating it toward the null, and several producing estimates in the opposite direction. *Take-home message: Differential residual confounding can generate marked between-site heterogeneity even when the underlying biological effect is constant, potentially creating the false appearance of effect modification or poor reproducibility across study sites.* **(B) Increasing bias in pooled estimates with growing cross-site confounding.** Here, each point shows the pooled regression estimate of the exposure effect from simulated multi-site data with increasing between-site differences in an unmeasured confounder. When sites are similar, the estimate is close to the true value (0.4, red dashed line). As site-level imbalance grows, the pooled estimate becomes increasingly biased, illustrating how large multi-site studies can yield very precise but systematically wrong inferences if cross-site confounding is not addressed. *Take-home message: Combining data from multiple sites without adequately accounting for site-specific differences can produce very precise but biased estimates.*

Larger sample sizes don’t reduce residual confounding. Conversely, they may increase its impact through greater precision. It is crucial to understand the provenance of the data, the structure of each site, the causal relationships, and variability between sites to avoid getting highly precise but biased findings.

## Harmonization and measurement error

Large datasets often rely on harmonizing data from multiple cohorts/sites collected under different designs, instruments, and time periods [41]. This data harmonization process allows investigators to merge variables into a single analytic framework and expand statistical power. Yet, harmonization introduces new layers of measurement error and information loss that can distort inference when unaccounted for [16,42]. For example, asthma may be defined through parental questionnaires in one cohort but through physician diagnosis in another; dietary data might come from 24-hour recalls on one site and food-frequency questionnaires in another. When these distinct sources are collapsed into a single “harmonized” variable, subtle differences in definition, timing, or instrumentation can alter the error structure and reduce validity. In large datasets where standard errors are tiny, even minor inconsistencies can yield results that are statistically convincing yet scientifically misleading [15,17].

Two broad types of measurement error emerge in large data research. Classical (random) error arises from imperfect precision of instruments within each cohort, typically biasing regression coefficients toward zero and masking true relationships. Systematic (differential) errors occur when measurement accuracy varies by cohort or participant characteristics—such as one site systematically under-reporting symptoms or using a different laboratory assay—producing biased results whose direction and magnitude differ by site [43]. The direction and magnitude of bias may be possible to estimate or approximate (if there is good insight into the design and data generation and collection process) or (most commonly) may be unknown and uncertain. When datasets with varying error structures are combined, these known and unknown biases can offset, amplify, or interact in unpredictable ways. Bias does not shrink with sample size: as precision increases, small structural errors become statistically significant, giving the illusion of confidence in biased estimates. Most large datasets represent routinely collected data rather than carefully defined prospectively collected information. Routinely collected data (e.g., insurance claims and EHRs) are not collected primarily for research purposes and this has major implications for measurement error that is often unrecognized and unprobed.

A further concern is harmonization asymmetry, the tendency to reduce variables to the lowest common denominator so that all cohorts can contribute data. For instance, a detailed maternal-diet score in one study may be collapsed into a binary “adequate vs. inadequate diet” indicator to align with other cohorts that collected only a few items. This strategy increases sample size but decreases measurement quality, trading validity for inclusiveness. Analysts often treat the harmonized variable as perfectly comparable across sites, ignoring that the underlying data quality differs. This can dilute or obscure true associations [44].

Analytically, these problems require either new capture of information that is standardized across all cohorts or sites, or when, typically, this is unfeasible or prohibitively cost-intensive, the use of models that explicitly recognize the diverse measurement processes. Approaches such as regression calibration [45,46], simulation-extrapolation (SIMEX) [47], latent-variable modeling [48,49], or multiple-indicator models [50] (see Box 1: Glossary) can correct or quantify bias when validation subsamples or repeated measures are available. Harmonization protocols should routinely document measurement error and cross-site comparability, treating these as sources of structured uncertainty rather than technical noise. A dedicated “measurement error workstream” that evaluates consistency across cohorts may prevent misleading precision in pooled analyses.

For some research questions, it should simply be acknowledged that large datasets cannot provide a valid answer. In several circumstances, additional, suitable data collection is not feasible and there is no data from validation subsamples or repeated measures. In other cases, a causal diagram may not be possible to specify with any reliability, or confounders may not be possible to measure. Investing then in tortuous analyses may be just research waste and may propagate misleading findings.

Figs 4–6 collectively illustrate how harmonization-induced measurement error may affect statistical inference in large datasets. Fig 4 evaluates agreement between site-specific and harmonized measurements using a Bland–Altman plot (see Box 1: Glossary). Although the mean difference is close to zero, indicating minimal systematic bias at the population level, the wide limits of agreement reveal substantial individual-level variability introduced by harmonization. Fig 5 illustrates attenuation from harmonization-related measurement error. We simulated a latent true measurement and generated an outcome with a true association of β=0.70. We then created a relatively precise site-specific measure and a noisier harmonized measure. Regression using the harmonized measure produced marked attenuation of the estimated slope toward the null compared with the true and site-specific measures. This demonstrates that harmonization-related measurement error can substantially weaken observed associations and make meaningful effects appear nearly null. Fig 6 generalizes this phenomenon by showing that attenuation becomes progressively more severe as measurement error increases. Overall, harmonization-related variability, even in the absence of systematic bias, can substantially distort inference.

**Fig 4:**
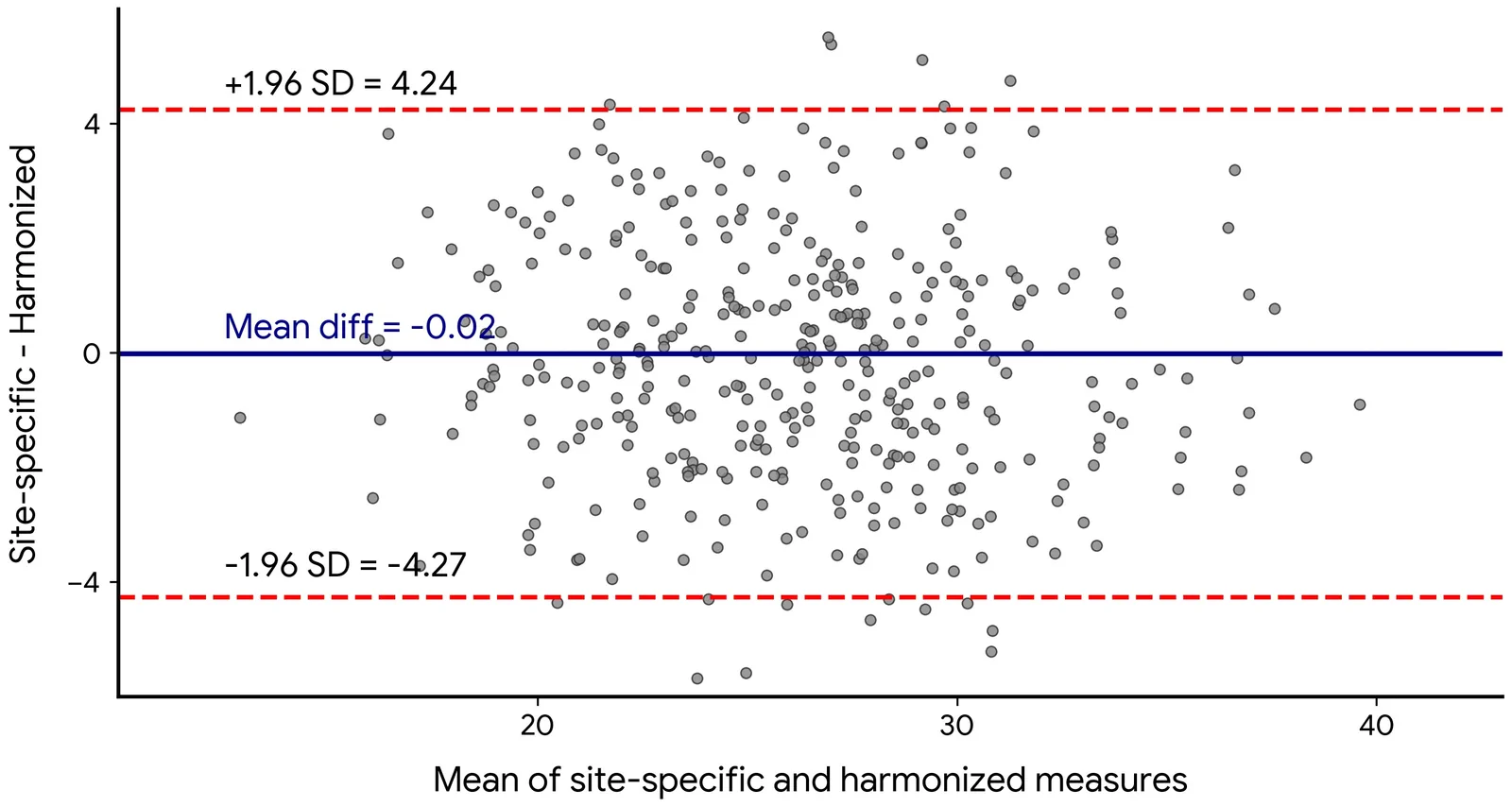
Agreement between site-specific and harmonized measurements (Bland–Altman plot). The Bland–Altman plot displays the difference between site-specific and harmonized measurements against their average. The solid blue line represents the mean difference (−0.02), indicating minimal systematic bias. The red dashed lines represent the 95% limits of agreement (−4.27 to 4.24), showing substantial individual-level variability. *Take-home message: Despite negligible average bias, the wide dispersion indicates that harmonization introduces considerable random measurement error.*

**Fig 5:**
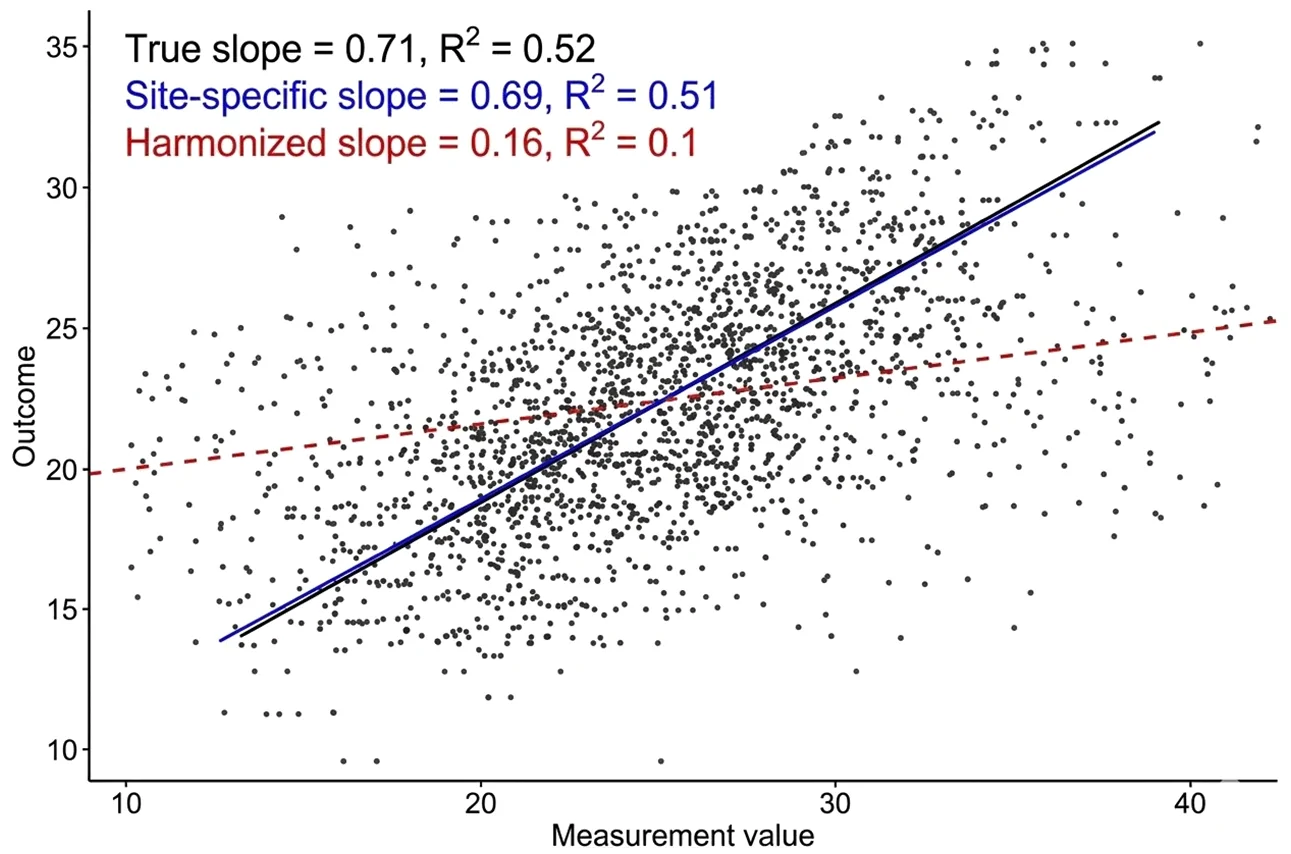
Attenuation of the exposure–outcome association using harmonized measurements. We simulated a latent true exposure for 800 individuals and generated an outcome with a true linear association of β=0.70. Two observed versions of the exposure were then created: a relatively precise site-specific measure with minimal error and a harmonized measure containing substantially greater measurement error to mimic imperfect cross-site harmonization. Separate regression models were fit using the true, site-specific, and harmonized measurements as predictors of the outcome. While the site-specific measure closely preserved the true exposure–outcome association, the harmonized measure produced marked attenuation of the estimated slope toward the null, reducing the observed association from approximately 0.70 to near 0.10. *Take-home message: Harmonization can introduce sufficient measurement error to substantially dilute true associations, making clinically meaningful effects appear weak or nearly absent.*

**Fig 6:**
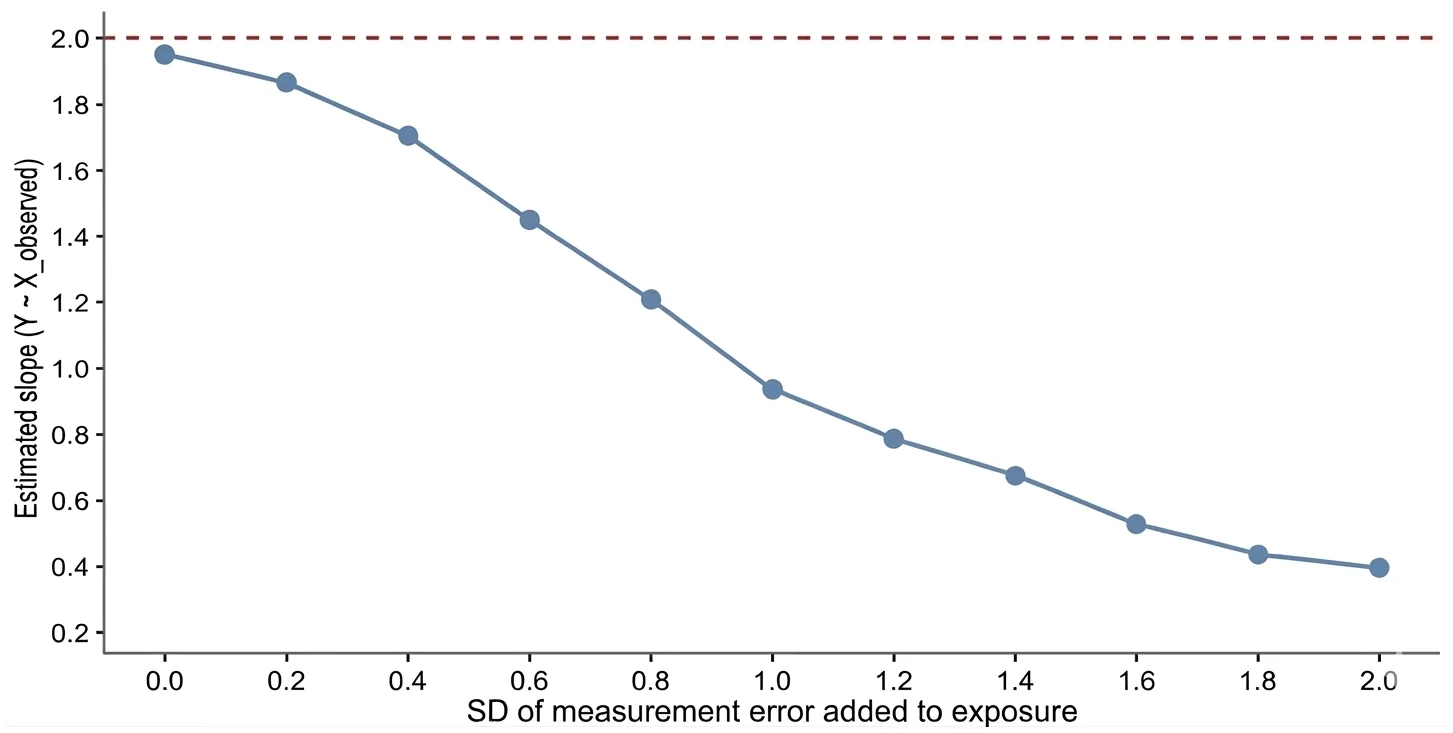
Attenuation of the exposure–outcome association with increasing measurement error. The red dashed line marks the true slope (2.0). As the standard deviation of measurement error added to the exposure increases, the estimated regression slope decreases progressively toward zero. *Take-home message: Increasing measurement error systematically weakens estimated associations and important effects can be missed.*

## Causal identification in the presence of rich covariates

Large datasets often offer the potential to capture a comprehensive range of demographic, biological, social, and environmental variables, which may facilitate the identification of causal effects with exceptional accuracy. However, as the number of covariates increases, so does the likelihood of analytic misidentification. While theoretically, expanded covariate sets may provide improved control for confounding factors, they also heighten the risk of incorporating inappropriate variables such as mediators, colliders, or proxies that can distort the causal pathway being examined [7,12,14]. Causal identification is determined not by the sheer volume of data, but by the underlying causal structure linking variables. This structure may or may not be possible to pre-specify conceptually. In the absence of conceptual pre-specification, large-scale data alone may have little or no ability to inform about the causal structure.

In numerous large biomedical databases, analysts frequently consider in the adjustment process all available variables, often employing automated selection techniques such as stepwise regression, LASSO, or high-dimensional propensity scores (see Box 1: Glossary) [51–53]. While such models reduce residual confounding from observed variables, they can introduce new bias if they condition on variables that lie downstream of the exposure or that act as colliders. Adjusting for mediators (for example, inflammation biomarkers between pollution exposure and asthma) will remove part of the true effect while adjusting for colliders (for example, a clinic visit rate affected by both exposure and unmeasured health status) can open spurious associations. Large datasets can amplify this problem if they contain information on numerous colliders and mediators. Causal identification is not predicated on including more variables, but rather on choosing the right ones.

Stepwise approaches and LASSO are not in general recommended approaches for variable selection for causal effect estimation. Confounders might be only weakly associated with the outcome but strongly associated with treatment. Lack of statistical significance does not correspond necessarily to lack of confounder relevance. Specifying all potential confounders may be recommended for causal questions, but in practice this is tenuous to do rigorously and with pre-specified clarity.

These methodological challenges also affect other popular analytical approaches such as propensity score and weighting methods. Leaving out even a single major confounder results in residual bias that larger samples cannot correct [54]. The most principled approach is to use DAGs (see Box 1: Glossary), temporal reasoning, and domain expertise to guide covariate selection whenever feasible. However, in many high-dimensional or complex observational datasets, fully specifying a well-informed causal structure may be difficult, impractical or impossible. Multiple plausible DAGs or adjustment strategies may be defensible depending on assumptions and investigator perspective. Sensitivity analyses that compare effect estimates across minimal, plausible, and expanded covariate sets can further reveal how robust findings are to different causal assumptions [12,37]. Multi-analyst approaches [55], where multiple analysts are asked to attack the same question and come up with and implement their preferred analytical plan, may also be helpful to calibrate the performance of such analytical inroads in the real world.

As shown in Fig 7A, inclusion of the primary confounder improved causal estimation relative to the crude model, but progressively expanding the adjustment set yielded little additional improvement in the point estimate while substantially widening confidence intervals. This illustrates that identification is not monotonic with adjustment size: adding more covariates does not necessarily improve causal accuracy and may reduce precision when additional variables provide limited identification value. In Fig 7B, increasing sample size under exposure misclassification narrowed confidence intervals around biased estimates without correcting the underlying attenuation bias. Neither larger adjustment sets nor larger sample sizes guarantee more valid causal inference when systematic bias remains unaddressed.

**Fig 7:**
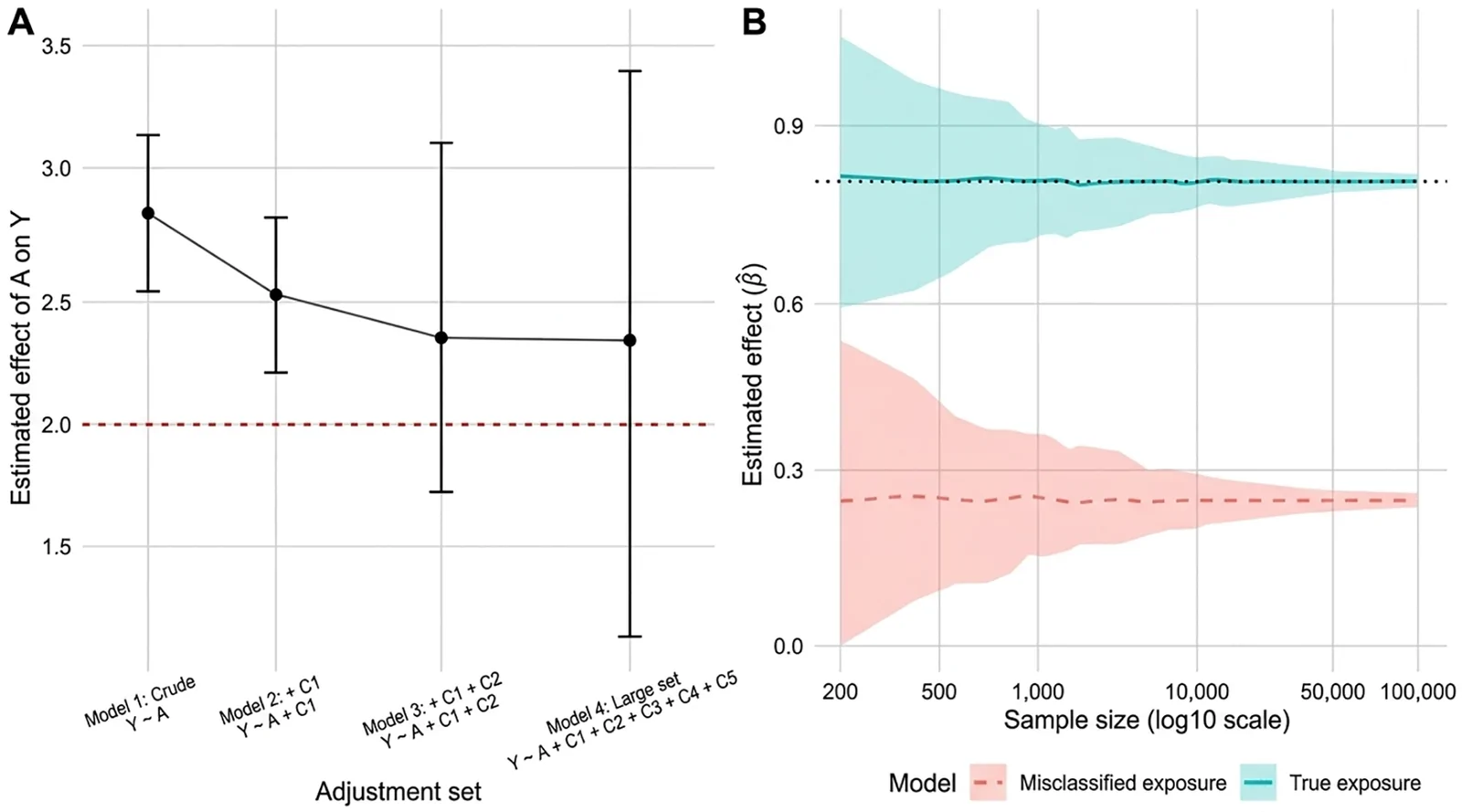
Larger adjustment sets and larger sample size do not guarantee better causal inferences. **(A) Non-monotonic bias from covariate adjustment in causal models.** Here, regression models were specified with progressively larger adjustment sets to illustrate that increasing the number of covariates does not necessarily improve causal identification. Model 1 included only exposure (Y~A); Model 2 adjusted for one key confounder ($Y\sim A+C_{\text{1}}$); Model 3 further adjusted for an additional covariate ($Y\sim A+C_{\text{1}}+C_{\text{2}}$); and Model 4 included a larger adjustment set ($Y\sim A+C_{\text{1}}+C_{\text{2}}+C_{\text{3}}+C_{\text{4}}+C_{\text{5}}$). The dashed red line denotes the true causal effect (β=2.05). Adjustment for the primary confounder reduced bias substantially, with the estimated exposure effect decreasing from 2.8 in the crude model to approximately 2.4 after covariate adjustment. However, further expansion of the adjustment set produced little additional change in the point estimate while progressively widening the confidence intervals. The increasing interval width reflects reduced precision from estimating additional parameters and potential multicollinearity/redundancy among covariates. Notably, the estimate remained biased above the true effect even after inclusion of five covariates, illustrating that larger adjustment sets do not guarantee unbiased estimation when key confounding structures remain incompletely captured, covariates are imperfect proxies for the true confounders, or residual/unmeasured confounding persists. *Take-home message: Causal identification may not be monotonic with adjustment size: adding more variables may reduce precision without necessarily improving validity.* **(B) Bias amplification with increasing sample size under exposure misclassification.** Here, we simulated a binary true exposure with a causal effect of β=0.80 on a continuous outcome and generated an observed exposure subject to substantial nondifferential misclassification (sensitivity = 0.60, specificity = 0.70). Regression models were fit using both the true and misclassified exposure across increasing sample sizes, with solid lines representing estimates based on the true exposure and dashed lines representing estimates based on the misclassified exposure; shaded regions denote empirical 95% simulation intervals across 300 replicates. As sample size increased, estimates based on the true exposure converged toward the correct effect, whereas estimates based on the misclassified exposure remained systematically attenuated despite progressively narrower intervals. *Take-home message: Larger sample sizes improve precision but do not correct systematic bias from measurement error, potentially creating false confidence in biased estimates.*

Together, these findings highlight a central challenge: the availability of numerous covariates does not guarantee valid causal inference. Instead, rich data environments increase the risk of incorrect adjustment, overfitting, and reliance on mismeasured variables. This may not be true if study design is principled (e.g., covariates prespecified based on assumed casual structure, or sample splitting procedures are used for predictive modelling). However, such principled designs are probably the exception in the literature.

## Practical guidance for inference in large datasets

The four methodological issues described above share a common theme: precision without structure does not guarantee truth. Large datasets expand analytic opportunity but may also amplify bias, shifting the primary threat from random error to systematic error. Consequently, analytic rigor and validity rather than reliance on statistical significance alone [5,7,9] are prime considerations. The following framework offers practical guidance for investigators working with large data.

1. *Decide where research fits within a continuum of exploration versus targeted causation*

Analyses should begin with a candid acknowledgement of whether one or more specific estimands are the goals of the research, as opposed to just probing massive associations in exploratory mode. If specific estimands are the goal, one should carefully assess whether one can meaningfully and cogently distinguish between total, direct, and mediated effects to anchor variable selection and clarify whether downstream variables (e.g., biomarkers on the causal pathway) should be adjusted for [7,56]. Often prior knowledge may not suffice to have such explicit, meaningfully targeted analysis planning. The range between a purely exploratory statistical exercise versus a carefully dissected scientific argument is very broad, and each investigation should be honest about where it lands in that spectrum [57].

2. *Evaluate both statistical significance and effect sizes in the proper context*

Since *p*-values decrease as sample size grows, statistical significance should be considered alongside effect size, uncertainty, and relevance in biological or clinical settings [6,9]. The effect’s size compared to a minimally important difference is more valuable than *p*-values alone. Visual tools like *p*-value versus sample size plots or −log10(*p*) against effect size can show when significance is mainly due to sample size rather than true association, helping focus on meaningful effects instead of arbitrary thresholds.

3. *Model site heterogeneity and don’t ignore it (e.g., when a site is probably a confounder or there are differences in measurement processes between sites).*

Although no statistical approach fully resolves site-related heterogeneity, analysts should explicitly evaluate and account for site structure whenever possible. Potential strategies include incorporating site indicators or hierarchical modeling approaches, assessing exposure–site interactions, and conducting within-site analyses followed by meta-analytic synthesis when appropriate [11]. The goal is not to assume that any single modeling strategy perfectly corrects site differences, but rather to recognize, quantify, and transparently report heterogeneity instead of ignoring it.

4. *Treat harmonization as measurement modeling*

Harmonized variables are not always directly comparable between data sources, so harmonization should be treated as a measurement process with inherent error. Techniques like regression calibration or latent-variable modeling (see Box 1: Glossary) can reduce bias, while tools such as Bland–Altman plots and attenuation simulations (see Box 1: Glossary) assess agreement and inference impacts [15]. Because harmonization often simplifies variables, it is important to consider sensitivity analyses on higher-quality subsets and clearly report trade-offs.

5. *Consider the use of causal diagrams to guide adjustment, whenever feasible*

DAGs can guide covariate selection by identifying the minimal adjustment set required to sufficiently block backdoor paths [7,14]. In large datasets with many correlated variables, algorithmic selection procedures typically cannot distinguish confounders from mediators or colliders. Causal discovery procedures may sometimes recover and distinguish some of these relationships (confounders, mediators, and colliders), but in practice they may require multiple possible DAGs (e.g., see completed partially DAGs [58]) and these processes have been rarely applied successfully to-date. Explicit causal diagrams may help prevent inappropriate adjustment and clarify the assumptions underlying the analysis. Comparing estimates across nested models can further assess robustness to specification choices. If causal diagrams are not possible to pre-specify in an informative manner, this should be acknowledged.

6. *Check covariate balance, not just model fit*

Covariate balance diagnostics are crucial when using propensity scores or inverse-probability weighting. Standardized mean differences before and after weighting clearly show group comparability [59]. In large datasets, minor imbalances can cause significant bias due to high precision. Weight stabilization, trimming (see Box 1: Glossary), and site-specific models may often be used, with balance results reported alongside main findings.

7. *Embed sensitivity analysis and exploration of the robustness of results as standard practice*

Because systematic bias dominates uncertainty in large datasets, sensitivity analysis should be a standard component of all analyses. This may include measurement error sensitivity analyses (e.g., restricting to high-quality data), site-level sensitivity analyses (e.g., excluding influential sites), or even much broader options, e.g., consideration of all possible analyses in a given space (so-called examination of vibration of effects (see Box 1: Glossary) [60–62] or those considered reasonable, defendable options based on some explicit rationale (e.g., specification curve analyses (see Box 1: Glossary) [63]).These approaches shift inference from a single estimate toward a range of plausible values that reflect structural uncertainty.

8. *Report limitations structurally*

Limitations should focus on causal structure and measurement methods rather than generic sample issues. For instance, investigators might note, “harmonization required merging varied definitions, possibly weakening associations,” or “models used minimally sufficient adjustment sets to prevent bias.” These statements show structural inference and improve clarity.

9.*Consider falsification calibration of performance*

One may be able to assess the performance of specific datasets and specific analytical approaches against readily available gold standard results. For example, falsification endpoints may be used [64–66] where associations that are very well documented to be null or to have specific genuine effects in previous work with strong evidence are probed in the dataset of interest to see what results that dataset provides for them. If null associations give statistically significant signals, what statistical significance threshold would eliminate them? And are true effects detectable?

10. *Consider multi-analyst approaches*

Many datasets are attacked by many investigating teams, but each team may report fragments of what they analyze and may focus on different or partly overlapping questions. Coalitions of investigators may perform multi-analyst evaluations [55,67,68]: they can agree in advance on what the important question is that they want to address, and each team then produces their results independently. One can then compare the similarity/dissimilarity of the analytical paths, justifications, and results produced.

11. *Enhance sharing, openness, transparency, and documentation*

Protocol pre-specification, and full sharing of data and of algorithms may help build more reproducibility for large dataset analyses [69–71]. However, these are proxies or good science practices, not the end-product of research. Moreover, open sharing may lead to unaccounted multiplicity, unless there is good and complete documentation of which teams have attacked a given large dataset and how. For targeted and confirmatory analyses, protocol pre-registrations should include detailed statistical analysis plans. Prospective registration of observational studies can become more common [5,72,73]. Conversely, retroactive registration [74,75] is of dubious value.

12. *Consider encompassing analyses rather than fragmentation into least publishable units*

A worrisome trend is the increasing use and misuse of large datasets for the generation of trivial analyses that serve as least publishable units. Moreover, there is some evidence that use of artificial intelligence tools may facilitate the production of such minimally informative papers [76,77], or even massively produce fake papers [78,79]. Conversely, more informative analyses may generate comprehensive evaluations of each dataset, generating analytical atlases that become the foundation for further investigation [80].

## Conclusions

Large-scale datasets have broadened scientific research, but key statistical challenges remain. While more data increases precision, structural bias persists and may even worsen due to interacting methodological issues. The methodological challenges that we described are not independent; rather, they interact in ways that can amplify bias as data scale increases. Valid inference in the large data era depends less on the quantity of data and more on the quality of design, measurement, and careful reasoning, including causal considerations whenever possible and relevant. By shifting the focus from statistical significance to validity, interpretability, and robustness, biomedical research can better realize the promise of large data while avoiding the pitfalls of being precisely wrong.

## Abbreviations

DAG: directed acyclic graph
EHR: electronic health record
METRICS: Meta-Research Innovation Center at Stanford
NHST: null hypothesis significance testing
